# The work system of prehospital medication administration: a qualitative mixed methods study with ambulance professionals

**DOI:** 10.1186/s12873-025-01213-z

**Published:** 2025-04-05

**Authors:** Kristian Ringsby Odberg, Karina Aase, Eystein Grusd, Anne Vifladt

**Affiliations:** 1 Department of Health Sciences Gjøvik, Norwegian University of Science and Technology, Gjøvik, Norway; 2 Department of Research, Innlandet Hospital Trust, Brumunddal, Norway; 3 Centre for Resilience in Healthcare, Faculty of Health Sciences, University of Stavanger, Stavanger, Norway

**Keywords:** Medication administration, Prehospital care, Human factors, Ambulance services, Patient safety

## Abstract

**Background:**

The characteristics of medication administration within the prehospital setting are underexplored. Ambulance professionals operate under varied levels of responsibility, dependent on their training and collaboration with local emergency facilities and other medical personnel. Given the critical condition of many patients using these services and the challenging environments they operate in, the risk of adverse drug events is significant. The aim was to advance the knowledge of the medication administration process in the setting of ambulance services.

**Methods:**

A qualitative mixed-methods design was applied to examine the medication administration process among ambulance professionals in a Norwegian hospital trust. Data collection included individual semi-structured interviews with 11 ambulance professionals at three ambulance stations, complemented by 114 h of observations. Interviews and observations were guided by the System Engineering Initiative for Patient Safety (SEIPS) work system model, and data were analyzed using a combined deductive-inductive content analysis.

**Results:**

The medication administration process in the ambulance work system is condensed into three stages: preparation, administration, and patient transfer, primarily due to constraints related to time and available information. The medication administration work system is influenced by a set of eight interrelated categories. These include technological aspects such as workarounds necessitated by inadequate equipment, organizational dynamics such as the fluid delegation of tasks, physical environmental conditions that impact on decision-making, and personal factors such as collaboration in managing critical patient scenarios.

**Conclusion:**

Medication administration tasks in the ambulance service take place along a continuum involving physical, technological, and organizational factors that interact and continuously influence ambulance professionals in their everyday practices. The study highlights the need for enhanced medication administration processes in ambulance services through improved collaboration, training, technological usability, and organizational adaptability.

**Supplementary Information:**

The online version contains supplementary material available at 10.1186/s12873-025-01213-z.

## Background

Little knowledge has been established concerning the characteristics of medication administration in the prehospital setting, of which ambulance services constitute one of the main pillars [1]. Prehospital drug administration is different from that in hospitals, as ambulance professionals (AP) have different levels of delegation responsibility, depending on their professional training. Furthermore, they rely on close collaboration with local emergency clinics, hospital emergency rooms, emergency medical communication centers, and prehospital medical doctors.

Patients using prehospital services are often critically ill, with many requiring initial intervention or resuscitation prior to reaching the hospital. Coupled with the fact that these services are often provided in challenging environments, with few diagnostic resources available, and for patients of varying acuity, the potential for adverse drug events is significant. International variation in prehospital service provision, staff training and experience, and the scope of practice add further complexity [2]. Rigorous studies of the occurrence of adverse drug events in the prehospital setting are lacking, yet a few studies have reported rates ranging from 4 to 13% [1, 3–5].

Safe medication administration is associated with the 5 Rs (right patient, right drug, right route, right time, right dose) [6] and relates to the individual behavior of health care professionals. However, safe medication administration also lies within a broader system [7]. This means that medication administration comprises social and technical factors that are interdependent and interrelated. In this study, we apply the System Engineering Initiative for Patient Safety (SEIPS), a system approach that is useful for understanding the medication administration process in its specific context [8]. It positions the medication administration process in a work system in which persons, physical environment, tasks, tools and technology, and organization interact to produce specific outcomes. In prehospital services, interruptions, multitasking, and fatigue are associated with adverse medication administration events, and studies have identified workload and long evacuation times as risk factors [9]. Furthermore, the urgency of patient situations spans a continuum ranging from routine transport to medical emergencies, which may explain the variability in performance across the work system.

There is a need for studies that take a systems approach to understanding the complexity of medication administration in different healthcare contexts, including the prehospital setting. The aim of the current study is therefore to advance the knowledge of medication administration within ambulance services. The following research question guided the study:

How can the medication administration process in an ambulance service be described according to a work system approach?

The study is part of a larger research project (TEAM-AMB) in which we implemented a team training program in a Norwegian ambulance service to investigate its impact on medication administration, teamwork, and patient safety culture [10].

## Methodology

### Study design

A qualitative mixed-methods design [11] was applied to examine the medication administration process among frontline APs, with individual interviews as the primary data source and observations as the secondary data source. The COREQ (Consolidated Criteria for Reporting Qualitative Research) checklist [12] guided the reporting of the study methodology and findings (Supplementary file 1).

### Setting

In Norway, Emergency Medical Services (EMS) are publicly funded and organized by regional health authorities. The system includes ambulance services, staffed by paramedics or emergency medical technicians (EMT), and emergency medical communication centers handling emergency calls and dispatch. APs operate under national guidelines and collaborate with general practitioners, hospital physicians, and helicopter EMS for advanced care services [13].

Drug prescribing in prehospital settings is governed by national legislation and guidelines. APs administer medications based on protocols authorized by regional health authorities, while medications outside these protocols require physician approval. Physicians, including helicopter EMS personnel, have broader prescription authorities for advanced clinical interventions [13].

The TEAM-AMB research project was conducted across seven ambulance stations within a Norwegian hospital, including rural and urban stations. Urban stations operate five ambulances each, whereas rural stations operate three ambulances each. Together, these ambulance stations serve a catchment area of 150,000 inhabitants and handle approximately 20,000 missions annually. For this study, data were collected from one rural ambulance station and two urban ambulance stations.

### Sample

The sample consisted of frontline APs, including licensed EMTs who have completed a four-year vocational high-school education program. Paramedics have either an additional one-year full-time equivalent university education or a three-year university education at the bachelor level, and physicians have a six-year professional degree in medicine. The recruitment of participants was conducted by ambulance station managers. For the interviews, a convenience sample of 11 APs from the rural ambulance station and from two urban ambulance stations, consisting of six paramedics, three emergency medical technicians and two physicians, was selected. Observations took place at the rural ambulance station and one of the urban ambulance stations, and the researchers shadowed different pairs of APs during their shifts. The total number of APs observed was 19 and some of the participants were part of both observations and interviews.

### Data collection

The interviews and observations were carried out between January and May 2022.

#### Interviews

Individual semistructured interviews were conducted at the three ambulance stations included. The participants determined the interview locations and times that best suited them. An interview guide (Supplementary file 2) was developed, informed by the medication administration process [14] and the work system elements of the SEIPS model [8]. To refine the guide, a pilot interview with an emergency medical technician from a different hospital trust was conducted, leading to adjustments, including rephrasing questions to be more open-ended and reordering topics. The participants were approached face-to-face, and interviews commenced with an overview of the study’s aim, clarification of voluntary participation, and confidentiality assurance. The interviews ranged from 40 to 60 min, were digitally recorded via a dictaphone application, were transcribed verbatim by the researchers, and were anonymized before analysis. All the interviews were conducted by a researcher with firsthand field experience in ambulance work. The transcribed interviews were read, and consistency was confirmed against audio files by the other members of the research team.

#### Observations

The observations focused on medication administration within the ambulance work system. They were conducted during the daytime on weekdays, and field notes were transcribed immediately after the shift. APs were consulted for clarification as required during the observations. A structured observation guide, which was based on the 5 Rs of correct medication administration [6], the medication administration process [14], and the elements of the SEIPS work system [8], was applied (Supplementary file 3). A total of 55 h of observation were conducted at the rural ambulance station, and 59 h at one urban ambulance station. To avoid a potential Hawthorne effect in which APs tried to act more positively than they did during everyday practice, the observers sought to be unobtrusive during missions and any post-mission follow-up questions were neutrally framed [15]. Additionally, we had regular meetings throughout the data collection period to calibrate the findings and discuss the observations. There were no indications that the observations had such effects on the participants.

### Analysis

The data material underwent deductive-inductive content analysis, encompassing three phases: preparation, organization, and reporting [16].

In the preparation phase, the data were anonymized, transcribed, and thoroughly reviewed multiple times. They were repeatedly discussed with the research group to gain a common understanding.

In the organization phase, we independently coded interviews and observations deductively by sorting medication-administration meaning units in a categorization matrix according to predetermined themes based on the work system of the SEIPS model [8]. This deductive first stage of the analysis process facilitated an operationalization of the data material with belonging concepts needed to make sense of the medication administration process.

**Fig. 1:**
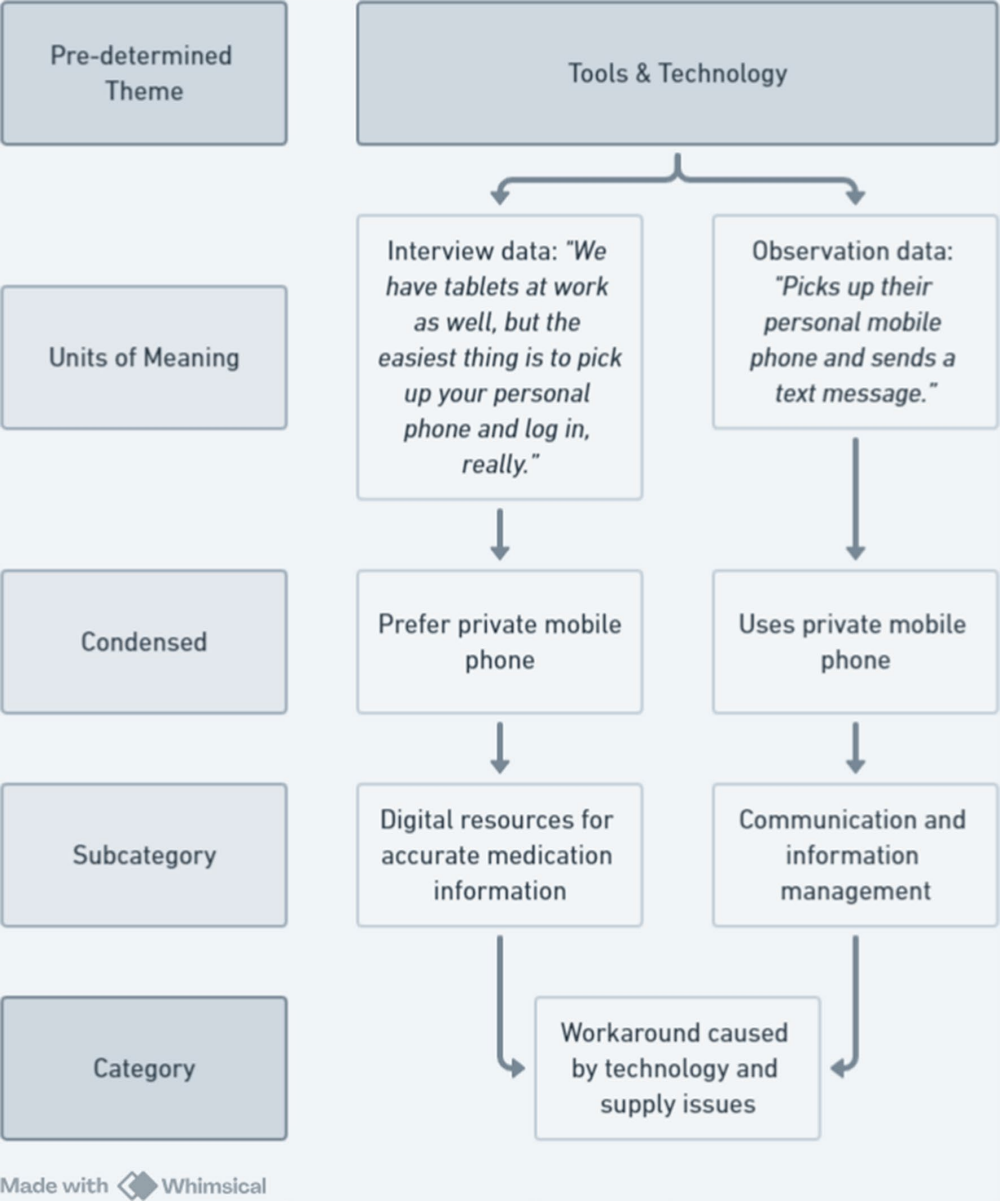
Example of the analysis process combining interview data and observation data

After completing the deductive sorting, we proceeded with an inductive analysis of the identified meaning units within each of the work system themes. Within each theme, we condensed the meaning units and identified descriptive subcategories (Fig. 1). The formation of subcategories was conducted separately for the interview data and the observation data. On the basis of similarities or differences across the two datasets, subcategories were then merged or eliminated to form the final categories within the themes. The observation data also led to a more general description of the APs’ medication administration work process.

**Fig. 2:**
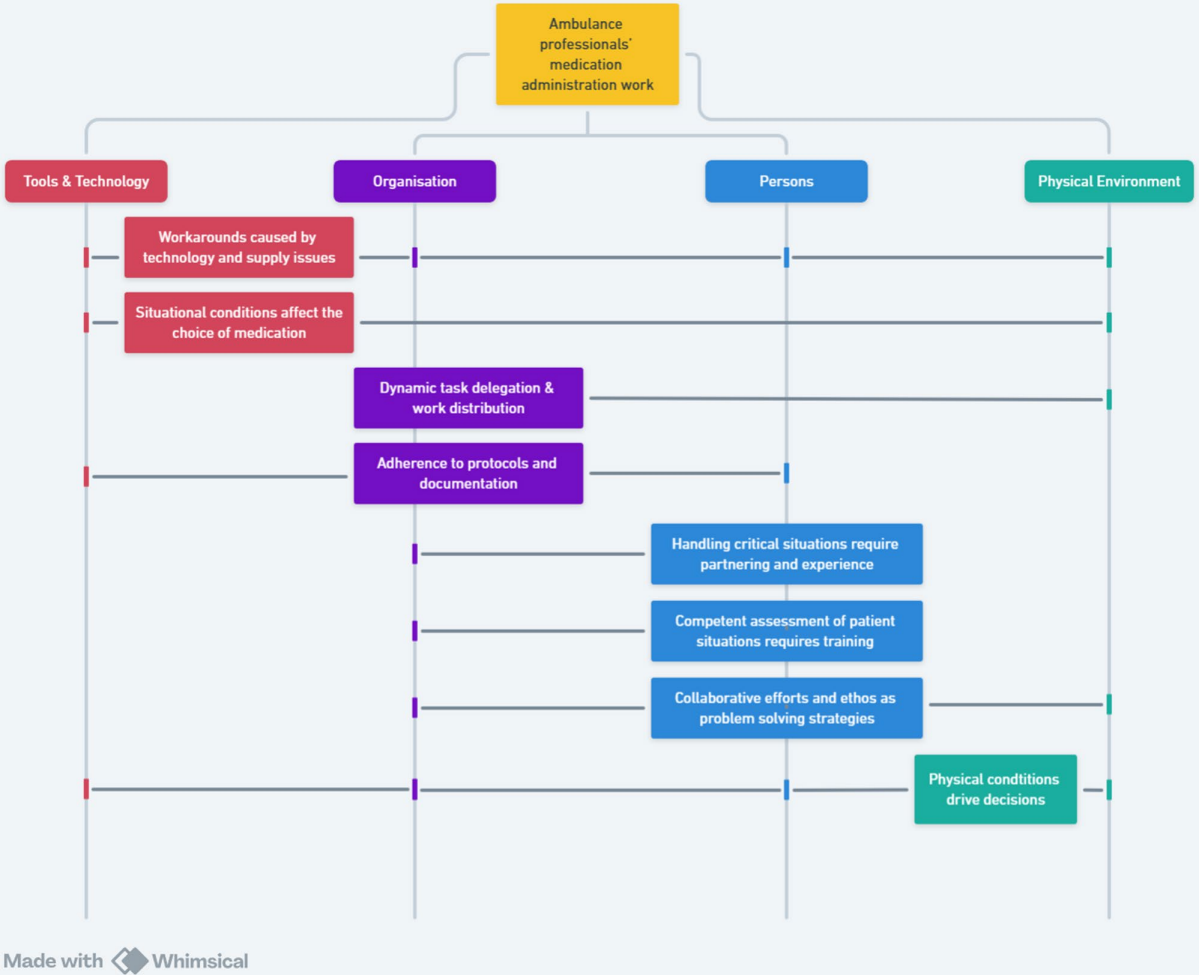
The work system of the medication administration process of an ambulance service

The results were presented as the work system of medication administration in the ambulance service comprising eight categories across four themes (Fig. 2).

### Ethics

The study received approval from the Data Protection Officer at the Hospital Trust (No. 16797830) and was reviewed by the Regional Committees for Medical and Health Research Ethics Central Norway (No. 250950). Additionally, written approval was obtained from the management of the prehospital division at the hospital trust to conduct observations of the employees in their everyday work.

The participants were provided with written information about the study and provided written consent to participate. The information outlined details about the study, emphasized the principles of voluntariness and confidentiality, and affirmed their right to withdraw from the study at any time without providing a reason. To safeguard the anonymity of participants, particularly in small ambulance stations, specific backgrounds were not disclosed.

## Results

Our analysis revealed eight categories within the four elements of the work system of the ambulance stations that significantly influenced the medication administration process (Fig. 2). The categories all interacted with and influenced the medication administration process in different ways. An example is how the choice of medication types and administration methods (tools & technology) was closely linked with the experience and competence of APs (persons), the use of guidelines and characteristics of collaboration (organization), and the locations where tasks were performed (physical environment).

### Ambulance professionals’ medication administration work

Medication administration is expected to adhere to protocols and regulations related to standing orders but is flexible in that APs use judgment calls and can contact receiving hospitals or associated physicians for advice. An example of such flexibility is how APs have individual preferences for certain types of pain medication for different types of injuries, based on prior experiences or familiarity with the medications.

Observations, clinical assessments and events in the ambulance, including medications, are documented continuously in the electronic patient journal. APs with delegated authority can administer a variety of medications on the basis of clinical assessment and their own judgment. The observations indicate that the medication administration process unfolds through three overlapping stages: preparations, administration, and patient transfer.

Preparing involves mental and practical tasks before and during callouts on missions, such as reviewing procedures and the proper dosages of potential medications, as well as retrieving actual equipment and medications. The APs often discuss likely scenarios and some flexible action plans upon arrival. Specific tasks are allocated on the basis of each team member’s expertise. One AP may be responsible for medication administration, whereas the other focuses on patient assessment. Their roles are usually defined based on their competencies and level of experience, while the final choice of medication is often discussed and agreed upon among them.

Medication administration unfolds onsite with the patient or within the ambulance where APs are responsible for accurate administration. Safe and proper administration of medication should adhere to the established protocols and guidelines. Whenever they are in doubt, APs consult a senior AP or a physician.

Patient transfer involves handing over the patient to a receiving unit, such as the emergency department, and writing a final report with all relevant documentation. An oral report follows, often highlighting critical information and recent patient record details.

The following sections describe the different themes in detail, using quotes from the individual interviews supported by descriptions of practice from the observations.

### Tools & technology

#### Workarounds caused by technology and supply issues

APs rely on a diverse array of technologies and tools to perform tasks related to medication administration. This spans practical equipment for medication-related tasks, communication tools, task distribution systems, and activities associated with documentation, reporting, and information transfer. Issues arise when equipment is not universally suitable for the various situations encountered, necessitating adaptability and the creation of workarounds by the APs.

Preceding and during missions, we observed how considerable time was dedicated to equipment preparation and medication checks. Uncertainty in the situations encountered often leads APs to predraw what they believe to be the correct medication. Concerns about making errors or selecting the wrong medication, especially when ampules appear similar, were frequently expressed.

*Yes*,* we are better at double controlling [medications] with our partner. We clarify what we can before we start driving. We think a bit further ahead than we did before. It might have something to do with the fact that the medicine bag used to be small; today*,* it is enormous. Today*,* we use medications much more frequently than we did before. (Interview AP1)*

APs occasionally faced challenges with missing or malfunctioning equipment. Instances were reported where the medication or equipment for administering the medication was either missing or had passed their expiration date. In most cases, these issues arose during normal preparations before callout, and it was easy to take corrective measures in due course.

Navigating the documentation system posed challenges, with numerous keystrokes required to access the correct information on their issued tablets. Some APs described issues related to touch functionality on tablets and small font sizes, making it difficult to see and press the right buttons. Consequently, they occasionally worked around this by postponing documentation until post mission, or when time allowed.

*You don’t see what it is you’ve written*,* and the fact that they* [letters and symbols] *can’t be blown up larger and adjusted*,* I don’t understand that. Additionally*,* there would be vibrations when driving*,* right? No chance! (Interview AP 2)*

Temporary notes were often jotted down on arms, notepads, or gloves, awaiting transfer to the electronic patient journal. Completing a journal or approving double checks relies on a dual signature with a four-digit code, which was occasionally shared among APs due to time and physical constraints.

#### Situational conditions affect the choice of medication

Medication management, in terms of both drug choice and administration route, was influenced by the conditions of the given patient or the task situation. Some APs suggested that a variety of available choices could lead to challenges, proposing a narrower selection and predosing options for certain medications, such as adrenaline and opioids.

*You know*,* dealing with elements such as bad weather and dim lighting all make giving medications outdoors challenging. In addition*,* all the gear we use outside? It’s made by folks indoors. Honestly*,* I don’t think having a whole bag of different medications makes the services any better. (Interview AP 4)*

The use of calculators and checklists varied, depending on the AP’s level of confidence with specific medications. In acute situations, where procedures were protocol-driven and doses were predetermined, unfamiliar or nonstandard scenarios prompted the use of supporting tools. APs favored intravenous or intranasal administration for control over dosage and efficacy, especially in critical and time-sensitive situations. Establishing intravenous access was often a top priority. Challenges in estimating patient weight, which typically rely on experience-based approximations, were noted. The choice of medication for pain relief depended on the patient’s condition and perceived urgency, with ketamine preferred for fractures and morphine preferred for severe abdominal pain.

### Organization

#### Dynamic task delegation and work distribution

Emergency ambulance missions commenced with the allocation of personnel to different vehicles, a task undertaken by the ambulance station manager on duty. A strategic team of experienced and inexperienced APs, along with mixed competencies, was prioritized to ensure operational effectiveness.

*Some possess a significant amount of knowledge*,* whereas others have limited expertise. However*,* the challenge lies in being able to perceive or understand the practical applications of the various forms of knowledge. (Interview AP3)*

When a patient is en route, in-ambulance coordination of knowledge and expertise occurs based on the most likely scenario. Tasks, potential medications, and equipment preparation were reviewed mentally and verbally, and guidelines and specific medications were clarified. Furthermore, external collaboration with entities such as emergency departments and anesthesiologists was coordinated. Discussions often revolved around the level of treatment and patient destination.

Role allocation was essential in the collection of patient information; a structured approach involved multiple sources, including clinical examinations onsite, input from informal caregivers if present, and a review of documentation. Home care services, when present, were also considered a valuable source of information. Coordinating this information could be challenging, and it was necessary for one of the APs to have a complete picture.

#### Adherence to protocols and documentation

Adherence to protocols played a crucial role in medication administration, especially in situations with uncertainty or with limited experience with a specific medication. Some medications were administered regularly, allowing APs to draw from personal experience, in addition to protocols. This was particularly valuable for opiate administration. APs with less experience relied heavily on written protocols and seldom deviated from prescribed dosages. Suspected diagnoses such as heart attacks and strokes often follow predetermined algorithms.

High levels of trust in one’s partner could sometimes lead to inadequate execution of double-control procedures. Several of the experienced participants described how they were sometimes less inclined to read the medication ampule labels or perform double control according to the protocol.

*For double control*,* it* [the quality] *is probably much better*,* the less experience you have. (Interview AP 8)*

Instances occurred where double control involving two APs independently verifying medication before administration could be challenging or absent, as in high-stress emergency situations with limited personnel or time constraints. APs could be required to administer medication quickly to stabilize a critical patient, and the presence of a partner for verification was not feasible. In such cases, APs had to rely on their training, knowledge, and established protocols to ensure accurate medication administration. In some prehospital settings, APs operate as solo practitioners without immediate access to partners. This lack of double control increased the responsibility placed on the AP to ensure medication safety. In some cases, the patient or their informal caregiver was asked to perform the double control.

*I have actually asked the patient to read on the ampule itself. What does it say here? It can also be a good way to do it because the patient does not truly know what should be written on the ampule. Therefore*,* at least I know that the patient has read it correctly. (Interview AP 2)*

The participants emphasized the importance of accurate electronic patient journal documentation, acknowledging the difficulty when entries were made retrospectively. In such cases, notes and verbal communication were essential for verification. In handover situations, such as those in the emergency department, disparate data systems necessitated physical printouts. Uncertainties also arose about who should receive the report, both in written and verbal forms, leading to instances of repeated verbal reporting and occasional misunderstandings.

### Persons

#### Handling critical situations requires partnering and experience

APs normally team up with regular partners who, over time, become familiar with each other’s strengths and weaknesses. In certain situations, APs ask for assistance from a range of persons and professionals, such as onsite home care nurses, patient relatives or other emergency personnel at an accident site. All the APs described that it was easier to work with people they already knew, reducing the need for ongoing communication. Delegating tasks and collaborating in critical situations then requires less visible and explicit communication.

In critical emergency situations, experience was a crucial element enabling APs to make quick decisions without necessarily consulting a third party. In situations of uncertainty, most professionals preferred to contact a physician for clarification or a more experienced colleague, if available. During consultations, most of the APs experienced collaborative decision-making processes involving a thorough discussion of the patient’s situation before medication administration. Independent assessment and treatment on the basis of specific findings were crucial. They focused on treating symptoms rather than diagnoses. If a physician was involved, it was in an advisory role, and direct orders were rarely given.

*We are here to provide advice*,* and it is always the person attending to the patient who is responsible for both the treatment they administer and the reasons behind it. However*,* we strive to offer advice that assists in the situation. If I say you can administer 40 milligrams of morphine*,* I hope the individual reacts to that. Therefore*,* it is not a directive but rather guidance. (Interview Physician 1)*

#### A competent assessment of patient situations requires training

Many APs described attending regular courses for medication administration, usually in a digital format. Several expressed a desire for more collaborative practical exercises. Some could not recall the last time they received professional training, whereas most indicated that opportunities for skills training were available if needed. While the APs acknowledged the importance of the “5 Rs” for proper drug management, few of them could articulate the five components or their implications.

*Therefore*,* if I can speak freely*,* I believe that the training can improve significantly. It is undoubtedly safe*,* but there is much room for it to be more user friendly and more focused on patient safety. I miss a more comprehensive training on the various medications. I truly feel that I fall short on some of the medications. Therefore*,* instead of just an annual recertification with simple questions such as calculating and diluting Morphine*,* I miss a bit more*,* a deeper training on it. (interview AP 3)*

Different perspectives on training were observed between older APs and younger APs. Young APs with less experience often referred more directly to protocols, whereas senior APs relied on their experience and intuition. Young APs also expressed a need for continuous training to keep abreast in the field and to ensure adequate personal competence levels.

Emphasis was placed on the importance of knowledge and competence in understanding patients’ medical histories, including medication lists, and grasping the effects and interactions of various drugs. An example was given of how a patient experienced extensive discomfort without fever, highlighting the importance of knowing that the patient was on a steady dose of paracetamol.

#### Collaborative efforts and ethos as problem-solving strategies

When APs chose medications in each patient situation, they relied on experience, intuition, and the opportunity to discuss them with colleagues. They understood and accepted that other professional groups or services could have different protocols while striving to accommodate and adapt as effectively as possible.

If the APs disagreed, protocols were used, and in some cases, it was common to involve a third party, a senior professional, for consultation. Occasionally, ethical dilemmas concerning the level of treatment and the choice of medications were described. APs were sometimes reluctant to administer certain medications if they were unsure of their effects or lacked experience with their use. A typical concern mentioned by many APs was the treatment of children:

*Yes*,* in essence*,* the situations we are more uncertain about put demands on us*,* such as with children*,* for example. Dosages in these cases*,* children*,* and particularly ill children*,* are something we encounter infrequently… (Interview AP 6)*.

A prevailing ethos, as described by several APs, was a commitment not to administer a treatment to someone whom they would not want to give to their own family members.

*The ethics in what I do at work is basically that if I wouldn’t want to expose my mother*,* father*,* sister*,* or nephew to it*,* then I shouldn’t expose the patient to it either. (Interview AP 1)*

Post mission discussions often centered around what went well and what could have been done differently. This collaborative effort was typically initiated in the ambulance and continued informally in the ambulance station’s duty room. The debriefing and experience-sharing sessions involved reflections on various aspects of specific work tasks, often referencing other incidents and practice situations.

### Physical environment

#### Physical conditions drive decisions

Medication administration in the ambulance services occurs in a variety of physical locations. Preparation and treatment can take place in the ambulance, as well as at an outdoor accident scene during different seasons, or in the patient’s home. Various challenges in the physical environment can influence drug choices and administration methods. In some locations, it is easier to treat patients onsite before transporting them to a hospital. At other times, environmental factors such as weather conditions and temperature may necessitate the rapid entry of the patient into the ambulance before medication treatment.

*I usually say that ‘we enter people’s private homes’. It varies. It can be anything from outdoors and subzero temperatures to tropical temperatures*,* and it can be indoors. It can be dirty. I find it terribly cramped. It does not mean I do not do the job. This creates challenges. I would rather insert an IV*,* perhaps outside with the patient*,* where I can have access to both arms*,* compared with inside that ambulance where I only have access to the left arm. (Interview AP 3)*

Ambulances come in various sizes, but they are uniformly well equipped with modern technology, yet working conditions can be demanding. There is typically space for two APs, in addition to the patient, in a large ambulance but seldom more than one in the back seat with the patient. Inserting a venous catheter while the vehicle is in motion can be challenging, especially during high-speed emergency responses or in uneven terrain. Therefore, establishing venous access and initiating medication before the ambulance departs is a priority.

*To be on the move*,* breaking glass ampules and drawing up with needles*,* it’s okay if it’s perfect conditions*,* but it doesn’t take much turning or stopping before it feels uncomfortable. (Interview AP 7)*

APs described how, in some cases, it was crucial to provide pain relief and stabilize the patient before moving, prioritizing fast-acting medications that are easy to adjust and assess their effectiveness. For example, anesthesiologists mentioned the effectiveness of intraosseous treatment, whereas others discussed intranasal treatment; however, most preferred early intravenous access.

Different equipment is situated in the rear compartment of the ambulance, and at times, it may be necessary to unbuckle the seatbelt to locate the required equipment. Several individuals found the use of electronic patient records challenging in a moving vehicle, relating to issues such as small fonts and an impractical user interface.

Communication with a partner could also be challenging because of movement and noise. This was addressed partly by shouting out loudly and partly by using radio communication and, in some cases, via phone.

## Discussion

In this study, we described the medication administration process in an ambulance service, revealing that the work system is influenced by a set of eight interrelated categories. These include technological aspects such as workarounds necessitated by inadequate equipment; organizational dynamics such as the fluid delegation of tasks; physical environmental conditions that impact decision-making; and personal factors such as collaboration in managing critical patient scenarios. We observed that the medication process in the ambulance work system is condensed into three stages: preparation, administration, and patient transfer. Our findings resonate with a recent systematic review by Walker et al. [17], which highlights similar influences on medication errors in the prehospital paramedic environment, ranging from organizational factors to patient-related factors.

### Sociotechnical factors

Our findings indicate that the interplay between technology, tools, and the physical environment impacts medication administration in ambulances. The diversity of medical equipment and medications necessitates a high level of expertise and constant decision-making, influenced by the environment, whether at the scene or in the ambulance. Effective preparation, such as arranging equipment and starting medication before departure, is vital. Once the ambulance is enroute, the complexity of executing procedures increases significantly, as the conditions make delicate tasks such as withdrawing needles or navigating and documenting in electronic patient journals difficult. Additionally, performing procedures in transit may compromise APs’ own safety. The participants in our study advocated simplifying procedures and using fewer types of medication.

Walker et al. [17] contend that environmental factors such as noise and inadequate lighting may be challenging to mitigate, in addition to the diverse and intricate nature of patients. They advocate prioritizing interventions on aspects amenable to control, such as standardizing medication dosages, labels, and equipment, including prepackaged medications. Our findings suggest that while such standardization may enhance medication safety, it may not always align with the dynamic reality faced by ambulance workers, necessitating adaptability to resolve complex situations [18]. A variety of physical conditions were shown to drive APs’ medication decisions on missions in this study. Similarly, Becker and Hugelius [19] described how factors such as ambulance speed, driving patterns, and communication between the patient, APs in the back of the vehicle, and the driver can impact the patient’s medical condition and the provision of effective medical treatment during transport. The aspect of personal safety was further elaborated upon by Hallihan et al. [20], who highlighted cases where APs need to loosen their seatbelts or navigate around cables and tubes.

### Experience

Our study documented that less experienced APs tended to rely more on guidelines and to consult reference materials during callouts, whereas senior APs more readily made decisions based on their experience. Critically ill patients receiving extensive medical treatment at the back of an ambulance while in transit emphasize the necessity of thorough planning and adaptive thinking, particularly during extended patient transport [21].

Caring for children manifested the role of experience among APs. In line with Nordén et al. [22], most participants in our study reported the administration of medication to children challenging, due to infrequent cases and high stakes. APs address this by relying on guidelines, communication, and double control and by seeking advice. A systematic review supports these findings, noting multifactorial dosing errors due to a lack of experience in a stressful environment [23]. Hörberg et al. [24] highlight that uncertainty among ambulance workers is linked to a lack of experience and aligns with our findings concerning the importance of experience and support from colleagues among APs.

### Double control

The APs in this study acknowledged that the level of closeness with their partner occasionally led them to skip the double control of noncritical medications. APs also administer medications in situations where thorough double control is challenging or impossible due to factors such as constrained space in the back of the ambulance, a critical incident, or being alone or separated from their partner. Several participants in the study explained how they managed this challenge by verifying the medications themselves or by engaging patients or their relatives in the double-control process.

Research on the use of double control outside of emergency medical services has shown mixed results, with insufficient evidence supporting the effectiveness of independent double control in medication handling [25]. However, it is still recommended when used alongside other strategies [23, 25]. There is a need for further studies to translate these findings into the ambulance services.

### Documentation and the electronic patient journal

The electronic patient journal in the ambulance services included in this study did not communicate with, for example, the emergency department’s data system, which requires APs to print a written report for use in the patient handover. Dúason et al. [26] reported that while nurses and doctors valued detailed reports from APs for accessing information post handover, APs themselves were skeptical about their utility, leading to occasional neglect in writing reports, as confirmed by the emergency department staff, who noted incomplete or absent reports. Our participants reported how handover situations often varied in the emergency department and that they sometimes left the handover unsure of the quality of the information transfer.

### Limitations

This study has some limitations. The station manager was responsible for recruiting participants for the interviews and the observations, and their relationship with the APs may have affected the final sample. Our sample lacks representation across all professional groups within the ambulance service, notably excluding nurses working in ambulances in the role of paramedic. This omission could skew the findings, as nurses play an important role in prehospital care. Observations were carried out only during daytime hours (7 am–10 pm) on weekdays, and by omitting nightshifts and weekends, important issues related to the medication administration process may have been missed. Our research is geographically limited to one region of Norway, which, together with the relatively small sample size, may impact the study’s transferability. We believe that our mixed methods approach using a combination of direct observations and interviews mitigates this by providing a more complete picture of the work system for medication administration in the current ambulance service.

## Conclusion

The ambulance work system for medication administration can be described in eight categories that are interrelated and interact in various ways. APs’ medication administration work related to *tools and technology* included workarounds and decisions affected by situational conditions. Their work related to *organizational issues* was characterized by dynamic task delegation and work distribution, as well as differences in adherence to protocols and documentation. *The personal work categories* were related to partnering, experience, training, and collaborative efforts to conduct competent assessments and problem solving. *The physical environment* drove decisions according to physical location, ambulance layout, and equipment availability. These factors, together with technological limitations, present challenges to double control and patient transfer. Other interactions include how APs’ experience, competence, and ability to adapt to various situations reflect on their use of protocols and guidelines.

The implications of this study are related to collaboration and training, improving technological usability, and building organizational structures allowing for adaptability in the work system of medication administration in the ambulance service. More specifically, training should be provided concerning medication administration to children. Routines for double control and documentation should be revised according to their practicality in the ambulance setting. Emphasis should also be given to the importance of commencing the medication administration process during callout.

## Electronic supplementary material

Below is the link to the electronic supplementary material.

Supplementary Material 1

## Data Availability

Supplementary data for file 1 is uploaded. Supplementary file 2 through BMJ: https://bmjopen.bmj.com/content/bmjopen/suppl/2023/01/20/bmjopen-2022-067006.DC1/bmjopen-2022-067006supp002_data_supplement.pdf. Supplementary file 3 through BMJ: https://bmjopen.bmj.com/content/bmjopen/suppl/2023/01/20/bmjopen-2022-067006.DC1/bmjopen-2022-067006supp003_data_supplement.pdf.

## Abbreviations

AP: Ambulance professional
SEIPS: System engineering initiative for patient safety
